# CD24 regulated gene expression and distribution of tight junction proteins is associated with altered barrier function in oral epithelial monolayers

**DOI:** 10.1186/1471-2121-10-2

**Published:** 2009-01-13

**Authors:** Ping Ye, Mangala A Nadkarni, Mary Simonian, Neil Hunter

**Affiliations:** 1 Institute of Dental Research, Westmead Millennium Institute and Westmead Centre for Oral Health, Westmead Hospital, Westmead, NSW 2145, Australia

## Abstract

**Background:**

Control of intercellular penetration of microbial products is critical for the barrier function of oral epithelia. We demonstrated that CD24 is selectively and strongly expressed in the cells of the epithelial attachment to the tooth and the epithelial lining of the diseased periodontal pocket and studies *in vitro *showed that CD24 regulated expression of the epithelial intercellular adhesion protein E-cadherin.

**Results:**

In the present study, the barrier function of oral epithelial cell monolayers to low molecular weight dextran was assayed as a model for the normal physiological function of the epithelial attachment to limit ingress of microbial products from oral microbial biofilms. Paracellular transfer of low molecular weight dextran across monolayers of oral epithelial cells was specifically decreased following incubation with anti-CD24 peptide antibody whereas passage of dextran across the monolayer was increased following silencing of mRNA for CD24. Changes in barrier function were related to the selective regulation of the genes encoding zonula occludens-1, zonula occludens-2 and occludin, proteins implicated in tight junctions. More particularly, enhanced barrier function was related to relocation of these proteins to the cell periphery, compatible with tight junctions.

**Conclusion:**

CD24 has the constitutive function of maintaining expression of selected genes encoding tight junction components associated with a marginal barrier function of epithelial monolayers. Activation by binding of an external ligand to CD24 enhances this expression but is also effective in re-deployment of tight junction proteins that is aligned with enhanced intercellular barrier function. These results establish the potential of CD24 to act as a potent regulator of the intercellular barrier function of epithelia in response to local microbial ecology.

## Background

Mechanisms responsible for the maintenance of the epithelial barrier critical for normal function in the gastrointestinal tract have been incompletely understood [1], with increasing interest in the barrier function of mucosal surfaces. In the oral cavity the epithelia attachment to the tooth presents a particular challenge as this is the only tissue environment where the eruption of teeth effectively results in a permanent breach in the integrity of the integument. In the process of tooth eruption the remnant of the epithelium responsible for secretion of the organic matrix of enamel melds with a down-growth of the oral epithelium to generate the epithelial attachment to the tooth [2]. CD24 is selectively strongly expressed by the epithelial attachment to the tooth and by the epithelium lining of the lesion of chronic periodontitis. This antigen is recognized by auto-reactive serum antibodies in patients with chronic periodontal disease [3] and increased titres of antibodies reactive with CD24 peptide correlated with more favourable diagnosis, suggesting a protective effect [3]. CD24 is a heavily glycosylated peptide ligand for vascular P-selectin and is anchored by phosphoinositol linkage to lipid rafts within the cell membrane [4]. It has been shown to be a regulator of the chemokine CXCR4 [5] and CD24 mediates expression of cell adhesion molecules in B lymphocytes with evidence for a signaling function for the CD24 receptor provided by the regulation of apoptosis in B cell precursors by monoclonal antibodies reactive with CD24 [6]. Isoforms of CD24 expressed as 33–35 kDa and 30 kDa entities typically contain N-glycosylation patterns including α2,3-sialic acid groups as primary sites for recognition by the L1 transmembrane receptor [7] that is also expressed by the epithelial attachment to the tooth and that lining the lesion of periodontitis (unpublished data). Intra- and inter-cellular signaling occurring through interaction between CD24 and L1 could be modulated by lectin-like molecules such as the sialic acid binding protein Hsa from *Streptococcus gordonii*, an early coloniser in bacterial plaque [8], or by antibodies that recognize CD24 [3]. These ligands for CD24 have potential to either activate signaling through CD24 or perturb signals mediated by constitutive interaction between CD24 and L1.

The reactive epithelium associated with inflammatory periodontal disease has a number of features that distinguish it from stratified squamous epithelia in other sites in the body. These include cytokeratin [9] and involucrin [10] expression profiles that do not support a typical pattern of terminal differentiation, reduced expression of E-cadherin and perturbation of F-actin filament structure [10]. This epithelium supports the transmigration of neutrophil polymorphonuclear leukocytes as a critical element of defence against the adjacent microbial biofilm [2]. Immuno-pathological responses to bacterial antigens are considered to be central in the pathogenesis of the destructive disease of periodontitis where the normal structure of the epithelium is perturbed [10] with evidence that cytokines released by adjacent inflammatory infiltrate cells act to disrupt epithelial tight junctions [11] responsible for barrier function to exclude low molecular weight microbial products.

The current consensus model for lining epithelium indicates initial cell-cell adhesion mediated by the cadherin complex as a key pre-requisite for the establishment of other types of cell junctions and of epithelial polarity; a property initially assigned to highly aligned epithelia such as the intestinal lining but more recently recognised to occur to a degree in most lining epithelia including the epidermis [12] and non-keratinised oral epithelial tissues [13,14]. The presence of tight junctions in normal stratified epithelia, metaplastic stratified epithelia and cultured derivatives of these tissues, has been a subject of controversy [14]. Functional studies have indicated the importance of claudin-1 expression in the permeability barrier of the epidermis of newborn mice [15] and Langbein *et al*. [14] have described a range of morphological types of close intercellular contacts associated with localisation of tight junction proteins in diverse mucosal sites.

E-cadherin association with the actin cytoskeleton is mediated by catenins and this complex also localizes the partitioning defective par-3/par-6/atypical PKC polarity complex [16]. Activation of this complex mediated by binding to the small GTPase Cdc42 results in phosphorylation and activation of atypical PKC. This pathway is considered to be important in promoting the appropriate formation of tight junction complexes possibly initiated by the binding of zonula occludens-1 (ZO-1) and zonula occludens-2 (ZO-2) to catenin. ZO-1 and ZO-2 function as sub-membrane anchors for the tight junction components occludin and the claudins [17]. Components of the intercellular junction structure include occludin [18] and the claudins comprising an extensive family of twenty four proteins [19]. The claudin composition of the tight junction defines the particular properties of the junction [20] with claudins-1, -2, -3, -4 and -7 reported to be prominent in stratified epithelia [21].

In the culture model used in previous study [22], H413 oral epithelial cells responded to specific suppression of CD24 mRNA and the associated reduction of surface CD24 protein by down-regulating *e-cadherin *expression. Ligation of CD24 on H413 cells by monoclonal antibodies to CD24 peptide resulted in strong stimulation of E-cadherin mRNA expression [22]. The objective of the present study was to investigate the potential for CD24 to regulate barrier function of oral epithelial monolayers in order to define mechanisms underlying the physiological function of the highly specialized epithelium of the periodontal attachment as the initial component responsible for the microbial barrier function of the oro-gastro-intestinal tract.

## Results and discussion

### Anti-CD24 peptide antibody reduces passage of dextran across the epithelial monolayers

**Figure 1:**
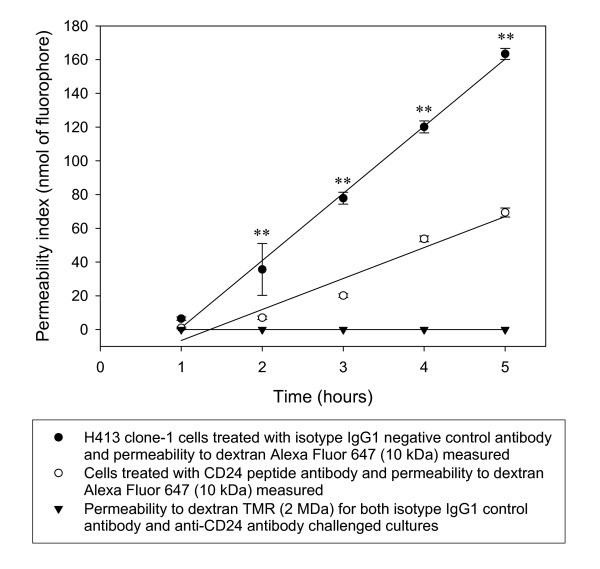
**Tight junction barrier function assayed in H413 clone-1 epithelial cell monolayers treated with anti-CD24 peptide antibody**. Antibody to CD24 reduces passage of fluorochrome-labelled dextran across H413 epithelial cell monolayers. Due to passage of dextran into the lower compartment reaching equilibrium after the 7^th ^hour, the rate of transfer was calculated over the first 5 hours. Regression analysis representative of three independent experiments for time points from triplicate cultures (showing mean values ± s.e.m.). Cultures were permeable for the translocation of low molecular weight dextran (Alexa Fluor 647 10 kDa, ** refers to *P *< 0.01, paired t-test), but were impermeable to high molecular weight dextran tetramethylrhodamine (TMR, 2 MDa).

Monolayers were cultured to confluence on membranes within diffusion chambers as described in Methods. The accumulation of labeled dextran in the lower chamber measured at time points showed H413 monolayers were impermeable to high molecular weight dextran tetramethylrhodamine (TMR, 2 MDa), indicating the integrity of the monolayer but were permeable to low molecular weight dextran Alexa Fluor 647 (10 kDa). Relative to isotype control antibody, addition of anti-CD24 peptide antibody resulted in a significant reduction in the accumulation of low molecular weight dextran in the lower chamber (Figure 1).

### Altered gene expression of tight junctions mediated by anti-CD24 peptide antibody

**Figure 2:**
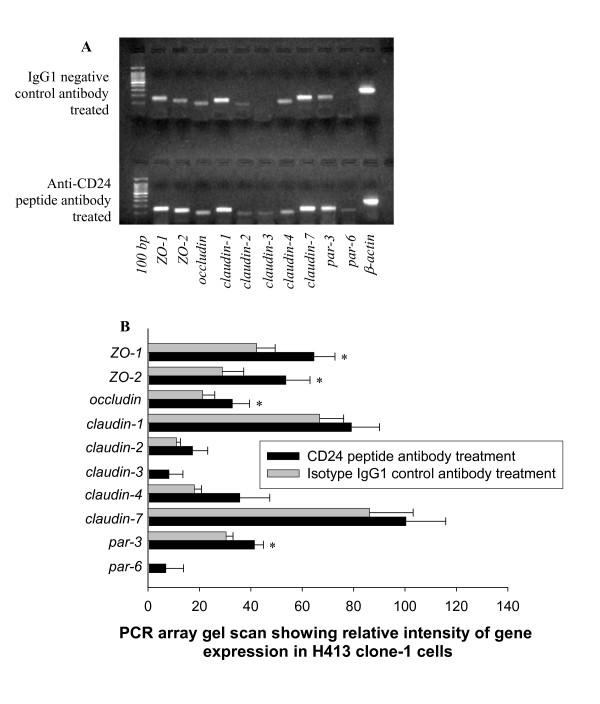
**Reverse transcription (RT)-PCR arrays for gene expression of tight junction proteins in response to challenge of H413 epithelial monolayers with anti-CD24 antibody**. (A) H413 clone-1 cells challenged with anti-CD24 peptide compared to isotype IgG1 control antibody for RNA extracted 3 h after addition of antibody. RT-PCR (30 cycles) reaction products were visualized on a 2% agarose gel after staining with ethidium bromide (representative of three separate experiments). (B) Bar graph showing mean values from three independent experiments (± s.e.m.) for anti-CD24 peptide antibody studies. The values for each experiment were normalized against the expression of β-actin (assigned as 100) and compared with the control sample and analyzed by paired t-test. * Refers to *P *< 0.05; four tight junction genes: *ZO-1*, *ZO-2*, *occludin and par-3 *showed significant differences that were confirmed by real-time quantitative RT-PCR analysis in Table 1. Expression of genes encoding claudin-3 and par-6 was not detected in control samples by real-time quantitative RT-PCR analysis.

Reduced transfer of low molecular weight marker to the lower chamber suggested tight junction function. The responses to anti-CD24 peptide antibody, of genes encoding tight junction proteins commonly expressed in stratified epithelia [21], were screened and analysed by RT-PCR arrays (Figure 2A and 2B). Significant up-regulation of genes encoding ZO-1, ZO-2, occludin and par-3 (Figure 2B) was validated by quantitative real-time RT-PCR (Table 1). Expression of *par-6 *and *claudin-3*, not detected in control cultures by 30 cycles of PCR, was demonstrable following challenge with anti-CD24 peptide antibody (Figure 2A and 2B). Gene expression for claudin-3 and par-6 was not detected in control samples by real-time quantitative RT-PCR analysis (Table 1).

**Table 1:** Real-time quantitative RT-PCR analysis of changes in expression of genes encoding tight junction proteins in response to anti-CD24 peptide antibody

**Gene**	**Ratio of average gene of interest (GOI): Test (CD24 peptide antibody treatment)/Control (IgG1 negative antibody)**	**Paired t-test** **P value (two tailed)**
*ZO-1*	1.98	< 0.05
*ZO-2*	1.5	< 0.05
*occludin*	1.24	< 0.001
*claudin-3*	Not detected in controls	
*claudin-7*	1.05	> 0.05
*par-3*	1.81	< 0.01
*par-6*	Not detected in controls	

### Changes in barrier formation of epithelial monolayers related to both increased levels and peripheral deployment of ZO-1, ZO-2, occludin and claudin-7

**Figure 3:**
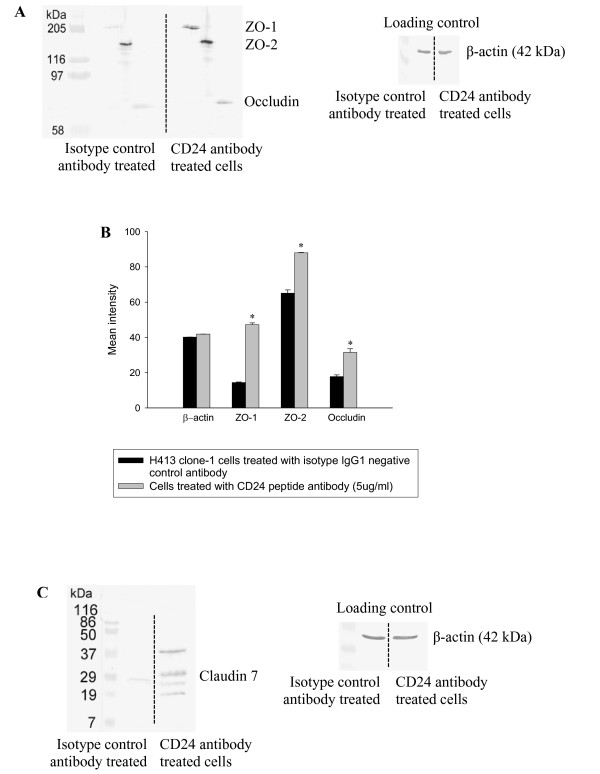
**Western blot analysis for representative tight junction proteins extracted from H413 clone-1 cell monolayers**. Data shown are representative of three independent experiments. Protein loading controls were confirmed by β-actin stain. (A) Showing increased expression of tight junction proteins ZO-1 (210 kDa), ZO-2 (160 kDa) and occludin (65 kDa) in cultures treated with anti-CD24 peptide antibody compared to H413 monolayers treated with isotype IgG1 (5 μg/ml) control antibody. (B) Bar graph confirmed increased expression of ZO-1, ZO-2 and occludin in anti-CD24 antibody treated sample compared to isotype control antibody by paired t-test (* Refers to *P *< 0.05). (C) Showing multiple bands in cultures treated with anti-CD24 peptide antibody for claudin-7 compared to H413 monolayers treated with isotype IgG1 (5 μg/ml) control antibody (see explanation: Results and discussion). Specific bands were not detected for claudin-3 or par-6 in either isotype control or anti-CD24 antibody-challenged culture extracts.

Cell lysates were probed with antibodies to tight junction proteins in Western blot analysis to confirm that the increase in gene expression was manifest as up-regulation of tight junction proteins. The data presented in Figure 3A and 3B indicate a significant increase in levels of ZO-1, ZO-2 and occludin in cultures challenged with anti-CD24 antibody. Par-6 and claudin-3 were not detected in either control or anti-CD24 treated cultures. Probing for claudin-7 indicated multiple bands in extracts of anti-CD24 treated cultures (Figure 3C) whereas a single reactive product was detected for cultures challenged with isotype control antibody. Multimers of claudins have been reported [23] and also complexes of claudin-7 with EpCAM, a pan-epithelial, homophilic intercellular adhesion molecule [24]. EpCAM was reported to form tight associations with claudin-7 that resist dissociation in SDS PAGE and result in a range of molecular weight entities detected by anti-claudin-7 antibodies [24]. It is possible that stimulation through CD24 induced complex formation of claudin-7 with EpCAM resulting in stabilization of the protein. This would explain the greater abundance of claudin-7 following stimulation with anti-CD24 antibody despite the lack of evidence for increase in mRNA expression for this product (Figure 2B and Table 1).

**Figure 4:**
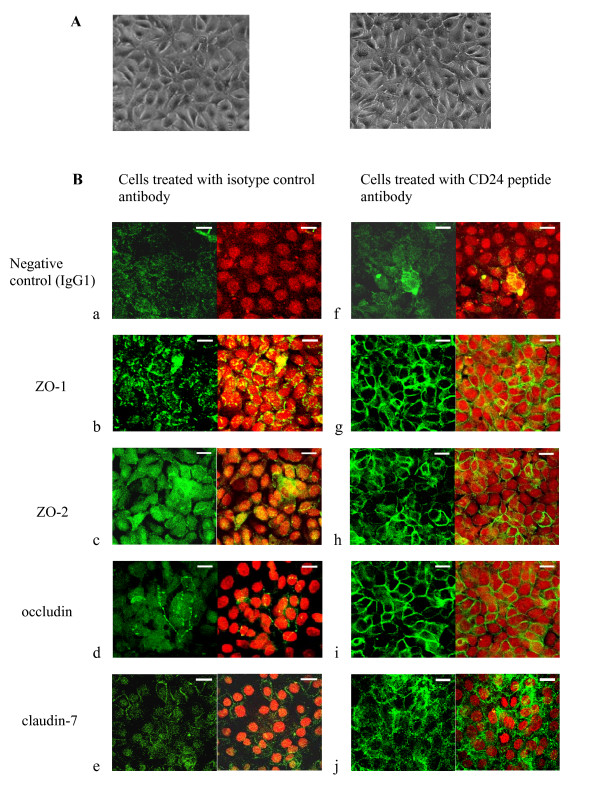
**Showing representative confocal laser scanning slice images for tight junction proteins**. Stimulation with anti-CD24 peptide antibody led to increases in tight junction proteins and altered cellular localization patterns indicative of functional tight junction complexes. Cells were grown on permeable Transwell filters until confluence as described in Methods. Data are representative of three independent experiments. (A) Phase contrast images showing confluence of cultures (cell density: mean value 3.2 × 10^4^/cm^2^) at 3 h after challenged with isotype control antibody (left) and with CD24 peptide antibody (right) prior to fixation. (B) Left panel shows confocal optical sections of representative monolayers that were challenged with isotype control antibody for 3 h before processing: (a) staining with mouse isotype control antibody; (b) tight junction protein marker ZO-1 showing focal staining principally at cell borders; (c) tight junction protein marker ZO-2 showing scattered cytoplasmic reaction; (d) occludin showing very weak reaction; (e) claudin-7 showing faint peripheral location. Right panel shows confocal optical sections of representative monolayer cultures that were challenged with anti-CD24 peptide antibody for 3 h before processing: (f) negative control show trace non-specific staining with mouse isotype control antibody; (g) ZO-1 well formed and localized at cell-cell contact; (h) ZO-2 expressed in 60 percent of the area of an optical section; (i) occludin showing peripheral distribution compared to trace cytoplasmic staining in monolayers exposed to control antibody (see d); (j) claudin-7 showing strong reaction that is diffuse near the cell periphery. This optical section image was captured at a deeper level. Claudin 3 and par-6 were not detected in isotype control or anti-CD24 antibody treated cultures. *Bars*, 20 μm. Note that the optical images were captured at a level corresponding to maximum staining in each case.

Confocal microscopy confirmed that while there was evidence from Western blot of moderate increase in tight junction protein expression in cultures challenged with anti-CD24 peptide antibody (Figure 3), there were marked changes in distribution from focal plaques or diffuse cytoplasmic staining in control cultures to continuous deposition at cell borders following challenge with anti-CD24 peptide antibody (Figure 4). Occludin, a component of the intercellular junction structure [18], was redistributed from a diffuse cytoplasmic pattern to a well-defined cell border pattern noted to be continuous by three hours after anti-CD24 antibody treatment. In contrast, ZO-1 was rearranged from a focal peripheral pattern to a continuous cell border pattern in keeping with its role as a sub-membrane anchor for tight junction components [17]. Although a similar role has been described for ZO-2, changes for this protein were less marked and the distribution pattern in cells challenged with anti-CD24 antibody was not uniform. The changes are compatible with protein kinase activity reported [25] to be implicatedin driving appropriate location of tight junction proteins. Claudin-7 showed both cytoplasmic and cell membrane localisation in cultures treated with either anti-CD24 antibody or isotype control antibody but with increased intensity of staining (Figure 4) in anti-CD24 challenged cultures.

Examination of the claudin-7 frame counter-stained with DAPI indicates that the claudin-7 network is located at a deeper plane as revealed by confocal optical sectioning. This finding could be explained by the report of localisation of claudin-7 to basolateral membrane complexes in association with tetraspanins [24] that are removed from the area of tight junctions [23]. While the present findings do not confirm a direct contribution of claudin-7 to the observed barrier function, membrane localised claudin-7 is reported to regulate E-cadherin expression [26], disclosing an indirect mechanism of support for effective junctions. Confocal imaging for par-6 and claudin-3 did not reveal the localisation of these molecules. These findings establish that stimulation by anti-CD24 induces selective up-regulation of components of tight junctions but more apparently, re-distribution of ZO-1, ZO-2 and occludin to the cell periphery where it is possible that the formation of functional tight junction complexes is responsible for the enhanced barrier function observed. The contribution of claudins, regarded as essential tight junction components, was not confirmed in this analysis.

### Distribution of F-actin supports the peripheral deployment of tight junction proteins

**Figure 5:**
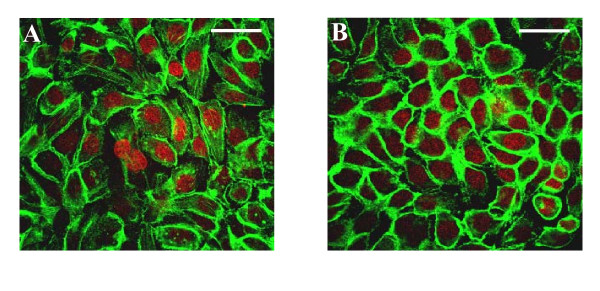
**F-actin re-distribution in response to challenge with anti-CD24 antibody**. Showing representative confocal optical slice images. (A) Cultures prepared 3 h after challenged with isotype control antibody. Showing variable profile from phalloidin staining. Some cells show typical F-actin filaments extending along the long axis of the cell. Others show clustering of F-actin to cell borders in regions of close intercellular contact. (B) Cultures prepared 3 h after challenged with anti-CD24 antibody. The majority of cells show intense staining for F-actin that is peripherally localized to cell borders. *Bars*, 20 μm.

Cultures treated with isotype control antibody demonstrated scattered cells with typical F-actin filaments extending through the long axis of the cell but with most cells displaying concentration of F-actin in regions of the cell periphery (Figure 5A). In contrast, cultures treated with anti-CD24 antibody demonstrated more uniform distribution with greater intensity of F-actin localised to the periphery of the cells (Figure 5B). The findings indicate a rapid and specific re-organisation of F-actin filaments in anti-CD24 antibody-treated cultures to support intercellular junctions.

### Ultrastructural analysis

Cross-sections of semi-thin sections stained with Toluidine blue indicated changes in morphology following challenge with anti-CD24 antibody. Cultures determined to be confluent by phase contrast microscopy, showed, at 3 h after incubation with isotype control antibody, mostly flattened cells with intercellular contacts localised to the region adjacent to the polyester membrane. Cultures challenged with anti-CD24 antibody contained a majority of cells with raised profile and more extensive intercellular contacts.

**Figure 6:**
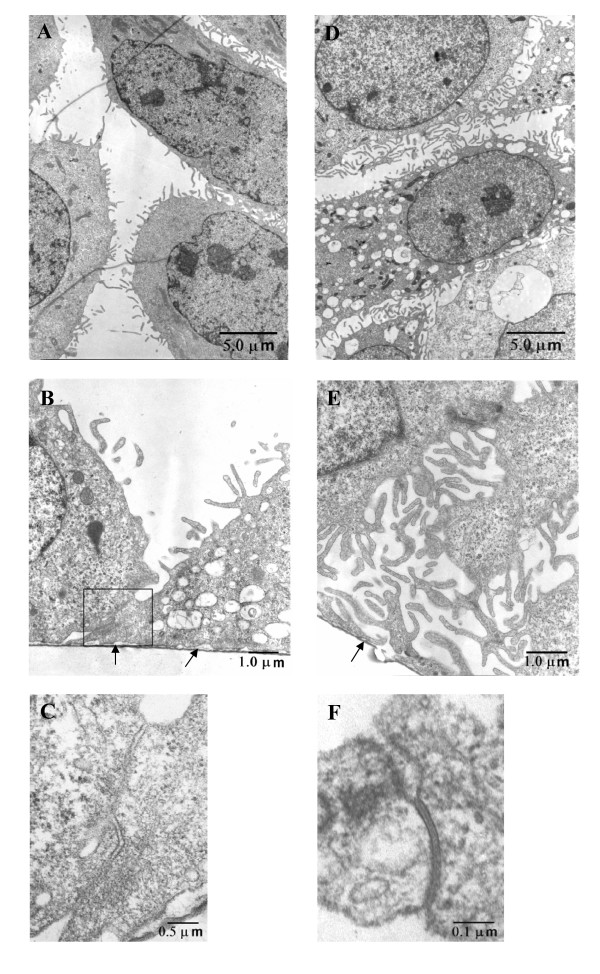
**Ultrastructural changes associated with response to challenge with anti-CD24 antibody**. Left panel showing culture fixed at 3 h after challenged with isotype control antibody. (A) *en face *section showing close intercellular contacts are mediated by cell processes that are not uniformly distributed. (B) Cross-section showing close intercellular contact confined to the basal region abutting the polyester membrane (arrows). (C) Showing higher magnification of long junction (box in B) with close membrane association. Right panel showing similar preparation of a culture challenged with anti-CD24 antibody. (D) *En face *section showing more uniform intercellular contacts mediated by cell processes. (E) Cross-section showing extensive intercellular communication mediated by extensive cell processes (arrow indicates supporting polyester membrane). (F) Showing tight junction-like intercellular contact.

Low magnification was used to evaluate the nature of intercellular contacts for those cultures challenged with anti-CD24 antibody versus isotype control antibody. It was observed for preparations sectioned *en face *that abundant cell processes were more uniformly distributed in cultures treated with anti-CD24 antibody (Figure 6D and Figure 6A). Close contacts in the basal regions abutting the supporting polyester membrane (Figure 6B and 6C) were evident in cross-sections of cutures challenged with isotype control antibody. In contrast, cultures treated with anti-CD24 antibody exhibited more extensive close contacts between processes of adjacent cells (Figure 6E). These contacts were characterised by intense staining of adjacent membranes, frequently in proximity to desmosomes. In cultures treated with anti-CD24 antibody, close membrane contacts included extended junctions (Figure 6F) characteristic of occludin-containing junctions in stratified epithelia and cultured derivatives of these tissues [14]. Similar structural entities were not detected in cultures treated with isotype control antibody despite frequent observation of regions of close intercellular contact (Figure 6C). Description of the distribution of proteins in the intercellular bridges of tight junctions of stratified mucosal epithelia has emerged only recently based on immunohistochemical and ultrastructural analysis [14,27].

### CD24 regulates transcription of genes encoding tight junction proteins in un-stimulated epithelial cultures

**Figure 7:**
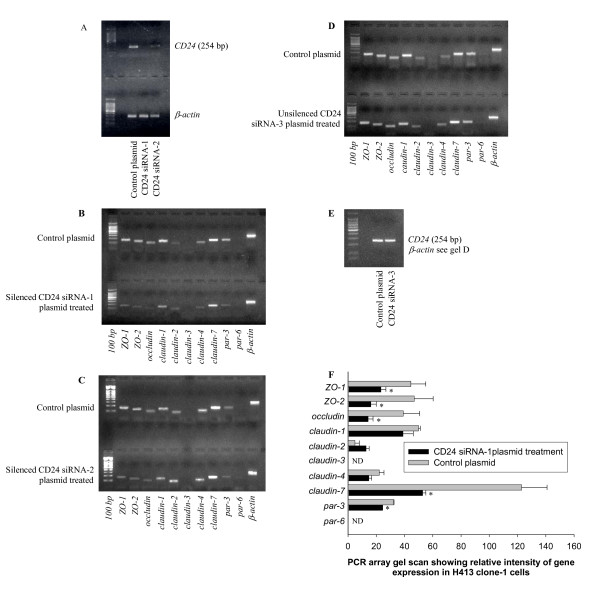
**Down-regulation of *CD24 *expression by siRNA is associated with reduced expression of selected genes encoding tight junction proteins**. Data shown are representative of three independent experiments in each case. (A) Showing down-regulation of mRNA for CD24 in H413 clone-1 cells with two different inserts compared to control plasmid following 30 cycles of RT-PCR. (B) Reverse transcription (RT)-PCR array (30 cycles) showing down-regulated gene expression of tight junction proteins for CD24 siRNA insert-1 silenced H413 clone-1 cells and compared to the cells transfected with control plasmid. Bands were visualized on a 2% agarose gel after staining with ethidium bromide. (C) Showing weaker suppression of expression of genes encoding tight junction proteins following transfection with the second CD24 siRNA insert that was less effective in suppressing *CD24 *expression (Fig. 7A). (D, E) Showing un-silenced CD24 following transfection by siRNA-3 (E) had no detectable effect on expression of genes encoding tight junction proteins as compared to plasmid control (D). [β-*actin *controls in D also apply to E]. (F) Bar graph showing RT-PCR arrays (30 cycles) showing gene expression in CD24 siRNA-1 silenced H413 clone-1 cells compared to plasmid control. Data are mean values from three independent experiments (± s.e.m.). The values for each experiment were normalized against the expression of β-actin (assigned as 100) and compared with the control sample by paired t-test. * Refers to *P *< 0.05; ND = not detected; five tight junction genes showed significant reductions that were confirmed by real-time quantitative RT-PCR analysis in Table 2. Expression of genes encoding claudin-3 and par-6 was not detected in either CD24 siRNA silenced cells or control plasmid culture samples by real-time quantitative RT-PCR. All experiments were performed 48 h after transfection.

**Figure 8:**
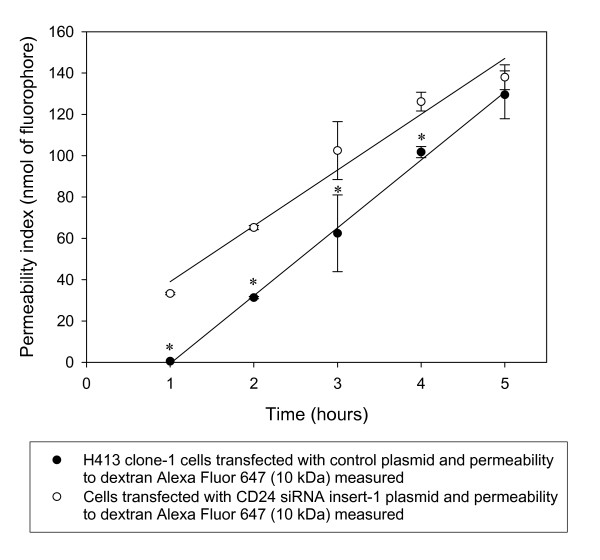
**Down-regulation of CD24 by siRNA is associated with increased epithelial barrier disruption**. Effect of silencing mRNA (insert-1) for CD24 in H413 epithelial monolayers. Regression data are representative of three independent experiments for time points from triplicate cultures (showing mean values ± s.e.m.). For experiments commencing 48 h after transfection, there is a significant difference between silenced and control cultures (paired t-test, * *P *< 0.05).

To determine if CD24 was important in maintaining basal levels of expression of genes encoding tight junction proteins an RNA interference strategy was employed as described in the Methods. Analysis of extracted mRNA indicated selective suppression of *ZO-1*, *ZO-2*, *occludin*, *claudin-7 *and *par-3 *expression (Figure 7B and 7C) that was confirmed by quantitative real-time RT-PCR (Table 2), with the degree of suppression correlating with the effectiveness of siRNA inserts in down-regulating CD24 mRNA (see Figure 7). This data indicated that CD24 functions normally to maintain levels of expression of selected genes encoding tight junction components. A potential mechanism is by signaling mediated by intra- and inter-cellular interaction of CD24 with L1 that is also expressed by these epithelial cells (data not shown). A high degree of inverse correlation was evident between those genes up-regulated by anti-CD24 treatment (Figure 2A and 2B) and down-regulated following silencing of *CD24 *expression (Figure 7B and 7F). Effective silencing of *CD24 *expression was associated with enhanced expression of *snail*, *twist *and *tgf-β3 *in this model [22]. Over-expression of Snail in an epithelial model also potently down-regulated expression of *occludin*, *claudin-3*, *claudin-4 *and *claudin-7 *accompanied by disruption of tight junctions and adherens junctions [28], providing a plausible link to explain the findings from the present study. Effective down-regulation of CD24 was associated with a short-term increase in permeability of the epithelial monolayer to low molecular weight dextran (Figure 8). This data suggested that CD24 maintains a minimal barrier function in un-stimulated cells.

**Table 2:** Real-time quantitative RT-PCR analysis of changes in expression of genes encoding tight junction proteins in response to down-regulation of CD24

**Gene**	**Ratio of average gene of interest (GOI): Test (silenced CD24)/Control (plasmid only)**	**Paired t-test** **P value (two tailed)**
*ZO-1*	-5	< 0.05
*ZO-2*	-2.27	< 0.01
*occludin*	-6.25	< 0.05
*claudin-3*	Not detected	
*claudin-7*	-1.3	< 0.05
*par-3*	-11.76	< 0.05
*par-6*	Not detected	

## Conclusion

In summary, CD24 levels observed in un-treated H413 clone-1 cells [22] are required to maintain moderate levels of expression of selected genes encoding tight junction components. This function did not support uniform peripheral deployment of the corresponding tight junction proteins but it was associated with a limited barrier function of the monolayer in restricting the passage of low molecular weight dextran. Silencing of *CD24 *expression was previously linked to down-regulation of E-cadherin [22]. Findings from the present study indicated the additional effect of down-regulating the expression of selected tight junction components could be responsible for the permeability changes observed. In this study, a monoclonal antibody to CD24 peptide was used to mimic the potential action of auto-reactive antibodies to CD24. The effect was to increase the synthesis of ZO-1, ZO-2 and occludin, but a more pronounced consequence was the re-deployment of these proteins to the cell periphery. For occludin, ZO-1 and ZO-2, the arrangement was compatible with tight junction formation whereas claudin-7 was distributed in a different manner. These changes were associated with substantial improvement of barrier function that was compatible with effective tight junctions. While the data indicate that CD24 has an important role in regulating expression of tight junction components, activation by an external ligand was necessary to induce a second function, the deployment of the proteins to the cell periphery associated with enhanced barrier function. The mechanisms for these two distinct functionalities remain to be explored.

## Methods

### Oral epithelial cell culture

The epithelial cell line (H413) derived from a human oral squamous cell carcinoma [29], displays stratified epithelial cell morphology in culture. H413 cell clonal lines were established using a limit dilution method as described previously [22]. H413 clone-1 cells exhibiting both characteristic epithelial morphology and high CD24 expression were chosen for this study. This clone was typical of more than half of the 50 clones that were successfully established indicating that it was broadly representative of the H413 cell line with respect to these characteristics. The cloned cells were cultured in Eagle's Minimum Essential Medium (MEM, Joklik modification, Sigma) and 5% fetal calf serum (FCS, CSL Limited, Victoria, Australia) {0.2 mM calcium contributed from FCS-[30]} at 37°C in 5% CO_2_. Cultures were harvested with 0.05% trypsin/EDTA in PBS and sub-cultured every 3 days.

### CD24 siRNA preparation and transfection of H413 clone-1 cells

The CD24 siRNA insert-1, 5'-TCCAACTAATGCCACCACCAA-3' (GenBank accession no. M58664), established as the most effective in specific suppression of mRNA for CD24 in H413 clone-1 cells was validated according to the protocol detailed in our earlier report [22]. This CD24 siRNA insert reduced CD24 mRNA in H413 clone-1 cells by an average of 90% and did not activate expression of the interferon response gene OAS1 [22]. The CD24 siRNA insert-2, 5'-GTCTCTTCG TGGTCTCACTC-3' was less effective (72–85%) in silencing CD24 mRNA. The CD24 siRNA insert-3, 5'-GCATCCTGAGCAACTCTTGAT-3' did not produce detectable silencing of CD24 mRNA level.

### The barrier function of epithelial cell monolayers

Barrier function of tight junctions in oral epithelial cells was measured by plating H413 clone-1 cells in 24 mm Transwell filters on 0.4 μm pore size polyester membranes (Corning Incorporated Life Science, USA). Briefly, 2.6 ml of medium was added to the lower compartment and 1.5 ml containing H413 clone-1 cells or cells transfected with CD24 siRNA or cells transfected with control plasmid, into the upper compartment. Cultures became confluent (cell density: 3.2 × 10^4^/cm^2^) by 48 hours when antibody (5 μg/ml) to CD24 peptide (IgG1, ALB9, Abcam, Cambridge, UK) which recognized a short non-glycosylated peptide sequence close to the site of GPI linkage of the peptide core of the cluster-w4/CD24 antigen [31] or the same isotype IgG1 negative control (DAKO, Denmark) was added to the upper chambers of randomly selected triplicate confluent monolayers together with low molecular weight dextran Alexa Fluor 647 (10 kDa Molecular Probes, Invitrogen) diluted 1:50 from a stock solution of 1 mg/ml in medium or high molecular weight dextran tetramethylrhodamine (TMR, 2 MDa Molecular Probes, Invitrogen) diluted 1:20 from a stock solution of 2 mg/ml. Dextrans were selected as bacterial products that are not taken up by epithelial cells and therefore are translocated by paracellular diffusion through intercellular junctions. At various time points (1–7 h, 9 h, 12 h) after commencing the experiments, 50 μl media were taken from each lower and higher compartment, and analysed for fluorescence using a Perkin-Elmer LS50B luminescence spectrometer, Ex650 nm/Em668 nm for Alexa Fluor 647, Ex555 nm/Em580 nm for TMR. Transfer of labelled dextran was calculated as moles of fluorophore transferred to the lower compartment determined by reference to a standard curve. Data from 3 independent experiments were analysed by paired t-test.

### RNA extraction and reverse transcription (RT)-PCR arrays

RNA extraction and RT-PCR was carried out as described previously [22]. The panel of target genes (see Table in Additional file 1) for tight junction proteins was selected on the basis of available literature for epithelia [21]. Primers were designed using Primer Express software (Perkin Elmer, Foster City, CA) and synthesised by Sigma. Expression profiling PCR arrays for the target genes were amplified in a thermal cycler (FTS-320, Corbett Research, Sydney, Australia) at 95°C, 15 min; 30 cycles of 95°C, 15 s; 60°C, 60 s. PCR array products (10 μl) from isotype control antibody challenged and anti-CD24 antibody challenged cultures were loaded into separate wells in a 2% agarose gel containing 0.5 μg/ml ethidium bromide in 1 × TAE buffer together with 5 μl of 100 bp DNA ladder (Promega). The images of the gels on a UV Trans Illuminator were captured by GeneSnap with CCD camera and analyzed by Gene Tools' software from GeneGenius (Syngene, MD, USA). Normalized values for relative intensity of genes of interest were determined by dividing each of the background-corrected RT-PCR band values by the background-corrected value for the β-actin housekeeping gene. Paired t-tests were used to analyse data from at least three consecutive experiments. PCR products were purified with UltraClean PCR Clean-up Kit (Mobio Laboratories, Inc., CA) and sequenced using respective forward primers (Westmead DNA, Sydney, Australia) to confirm sequence specificity of the amplicons.

### Real-time (TaqMan) RT-PCR for altered gene expression: ZO-1, ZO-2, occludin, claudin-3, claudin-7, par-3 and par-6

Real-time RT-PCR analyses were performed by TaqMan assays using the ABI PRISM 7700 Sequence Detection System and software (Applied Biosystems, Inc., Foster City, CA). The dual-labelled probes [shown in Additional file 1, Table] were designed using Primer Express software (Perkin Elmer, Foster City, CA) and synthesised by Applied Biosystems (probes labeled with fluorescent dyes 6-FAM at 5' end and TAMRA at 3' end). TaqMan PCR Core Reagent Kit and housekeeping gene β-actin kit were purchased from Applied Biosystems (ABI, Foster City, CA) as well. According to the kit protocol, 2 μl of diluted cDNA samples, 200 nM of each probe, and 200 nM of respective forward and reverse primers in a 25 μl final reaction mixture were used. The PCR reaction was initiated by activation of AmpliTaq Gold at 95°C for 10 min, followed by 40 PCR cycles: denaturation at 95°C for 15 s, annealing and extension at 60°C for 1 min. β-actin cDNA isolated from H413 clone-1 cells was used for constructing standard curves (2000-2 pg). For TaqMan assays percentage changes for target genes compared with β-actin controls were analysed by paired t-tests. A level of *P *< 0.05 was accepted as statistically significant.

### Western blots for ZO-1, ZO-2, occludin, claudin-3, claudin-7 and par-6

Following adjustment via loading controls proteins extracted in SDS sample buffer from H413 clone-1 cells treated with isotype control antibody or mouse monoclonal antibody to CD24 peptide (5 μg/ml) for 3 h, were separated by PAGE using 7.5% or 12% mini-gels, transferred to nitrocellulose membranes (Bio-Rad) and blocked overnight with 3% bovine serum albumin (Sigma) in 0.1 M Tris buffered salts solution pH 7.4 (TBS). Blotted antigens were incubated with mouse monoclonal anti-ZO-1 (1 μg/ml), ZO-2 (1 μg/ml), occludin (1 μg/ml), claudin-7 (1 μg/ml) antibodies (ZYMED Laboratories, Invitrogen) and mouse monoclonal anti-β-actin (0.2 μg/ml) loading control antibody (Abcam Ltd., Cambridge, UK) or rabbit polyclonal anti-claudin-3 (1 μg/ml), par-6 (1 μg/ml), and β-actin (0.1 μg/ml) loading control antibody (Abcam Ltd., Cambridge, UK) in 0.05% Tween20/TBS for 2 h, washed and incubated with alkaline phosphatase (AP)-conjugated second antibody (goat-anti mouse or rabbit IgG, DAKO, Denmark) diluted 1:1500 in Tween20/TBS for 2 h. Bound antibody was visualized with AP substrate (Bio-Rad) after development of reactivity for proteins from isotype control antibody and anti-CD24 antibody treated cultures under standardised conditions.

### Immunostaining and confocal laser scanning microscopy (CLSM) for ZO-1, ZO-2, occludin, claudin-3, claudin-7 and par-6

H413 clone-1 cells on Transwell filters (Corning) were grown to confluence (cell density: 3.2 × 10^4^/cm^2^) and were randomly assigned to receive either isotype control antibody or monoclonal antibody (5 μg/ml) to CD24 peptide for 3 h, washed in PBS then fixed with 4% paraformaldehyde/PBS for 15 min, permeabilized with 0.1% Triton-X100/PBS for 15 min, and blocked with 10% horse serum/PBS for 1 h. The filters were then cut into pieces and placed in micro tubes (1.5 ml), then incubated overnight at 4°C with rabbit anti-mouse IgG (Sigma) at 40 μg/ml to block binding sites on mouse IgG that could have remained attached to the cells as anti-CD24 peptide antibody. Following three wash cycles in 10% FCS/PBS, the preparations were incubated with primary antibodies: mouse monoclonal anti-ZO-1 (15 μg/ml), ZO-2 (1 μg/ml), occludin (2 μg/ml) and claudin-7 (5 μg/ml) or rabbit polyclonal anti-claudin-3 (5 μg/ml) and par-6 (5 μg/ml) in 10% FCS/PBS at 30°C overnight in a humid chamber. After washing with PBS, fluorochrome-conjugated second antibody (goat anti-mouse or rabbit FITC, DAKO), was added overnight at 4°C. For negative control, the primary antibody was replaced with isotype control antibody (DAKO). Membranes were washed with PBS and mounted onto glass slides using ProLong Gold antifade reagent with DAPI (Molecular Probes, Invitrogen).

Confocal images were captured with an Olympus Fluoview (FV) 1000, equipped with Olympus FV 10-MCPSU (405 nm, 473 nm, 633 nm) and NTT Electronic Optiλ (559 nm) lasers, at Westmead Millennium Institute confocal microscopy laboratory. Fields were selected at random [objective: Olympus 60×/1.20/0.28 (WD) Water UPLSAPO] and the cells were brought into focus under bright-field conditions. All fluorescence images prepared with confocal acquisition software (FV10-ASW 1.7) were stored and exported as TIF image files.

### Fluorescent phalloidin staining for F-actin

Cultures were established on Transwell membranes as described for studies of paracellular permeability. Confluent cultures (3.2 × 10^4^/cm^2^) were randomly assigned to receive either monoclonal anti-CD24 antibody (5 μg/ml) or isotype control antibody. After 3 h incubation cultures were washed in PBS. For F-actin staining, cells were fixed in 4% paraformaldehyde/PBS for 15 min, permeabilized with 0.1% Triton-X100/PBS for 15 min, and blocked with 10% horse serum/PBS for 1 h. Membranes were stained with FITC-Phalloidin (Sigma at 50 μg/ml) in FCS/PBS with DAPI nuclear counterstain and examined by confocal microscopy as described above.

### Transmission electron microscopy (TEM)

For TEM analysis, cells prepared as for F-actin stain were fixed in Karnovsky's fixative for 2 h followed by post-fixation in OsO_4 _for 1 h. Preparations were dehydrated in graded alcohols and embedded in low viscosity resin (TAAB Laboratory and Microscopy, United Kingdom). Semi-thin sections were stained in Toluidine blue to facilitate the selection of site and orientation for preparation of ultrathin sections in cross-section and *en face*. Sections were mounted on Pioloform/formvar coated slot grids, stained in uranyl acetate and lead citrate and examined in a Phillips CM10 electron microscope. Film negatives were scanned at 100% scale at 4000 pixels/inch using a Nikon Super Coolscan 8000.

## Authors' contributions

PY contributed to the experimental work and jointly drafted this manuscript with NH. MN provided oversight for the real-time RT-PCR studies. MS performed the immuno-histochemical studies with PY. NH participated with PY in study design and in writing up. All authors read and approved the final manuscript.

## Supplementary Material

Additional file 1 **The profile of tight junction genes**. The table provides the profile of tight junction genes expressed in H413 clone-1 gingival epithelial cells using RT-PCR arrays and real-time RT-PCR, including primers and TaqMan probes. Click here for file
