# Supplementary material for: CD24 regulated gene expression and distribution of tight junction proteins is associated with altered barrier function in oral epithelial monolayers
Source: BMC Cell Biol. 2009 Jan 13;10:2. doi: 10.1186/1471-2121-10-2 (PMC2632613; doi:10.1186/1471-2121-10-2)
Supplement: Additional file 1 — The profile of tight junction genes. The table provides the profile of tight junction genes expressed in H413 clone-1 gingival epithelial cells using RT-PCR arrays and real-time RT-PCR, including primers and TaqMan probes. [file 1471-2121-10-2-S1.doc]

**Additional file 1. The profile of tight junction genes expressed in H413 clone-1 gingival epithelial cells using RT-PCR arrays and real-time**

**RT-PCR**

| Tube | Gene Name | Expected size (bp) | UniGene Nos | GenBank Accession Nos | Oligos (sequence 5′ → 3′) |
| --- | --- | --- | --- | --- | --- |
| 1 | tight junction protein zonula occluden-1 (ZO-1) | 163 | Hs.510833 | NM_003257 | Forward AGAAGGATGTTTATCGTCGCATT  Reverse CCAAGAGCCCAGTTTTCCAT  Probe-Reverse TATCCACAACACGGAACACCTCTCCTTTG |
| 2 | tight junction protein zonula occluden-2 (ZO-2) | 130 | Hs.50382 | NM_004817 | Forward GGAAGGTCGCTGCTATTGTG  Reverse CGGAAACTTCTGCCATCAAAC  Probe CCAGCCCTCCCCTGGATCAGGAT |
| 3 | occludin | 98 | Hs.592605 | NM_002538 | Forward GTCCAATATTTTGTGGGACAAGG  Reverse GGCACGTCCTGTGTGCCT  Probe CACATTTATGATGAGCAGCCCCCCAA |
| 4 | claudin-1 | 135 | Hs.439060 | NM_021101 | Forward CAATGCCAGGTACGAATTTGG  Reverse TGGATAGGGCCTTGGTGTTG |
| 5 | claudin-2 | 81 | Hs.522746 | NM_020384 | Forward CTGCTTTTCCTGCTCATCCC  Reverse AGAGCTCCTTGTGGCAAGAGG |
| 6 | claudin-3 | 84 | Hs.647023 | NM_001306 | Forward ATCGTGTGCTGCGCGTT  Reverse GGCCCTCCCAGATGTTCTG  Probe-Reverse CCGACACGCGCCACATGGG |
| 7 | claudin-4 | 116 | Hs.647036 | NM_001305 | Forward TCATCGGCAGCAACATTGTC  Reverse GCAGTGCCAGCAGCGAGT |
| 8 | claudin-7 | 167 | Hs.513915 | NM_001307 | Forward CTCGAGCCCTAATGGTGGTC  Reverse CTACCAAGGTGGCAAGACCTG  Probe-Reverse CGATGAAAATTATGCCTCCACCCATGG |
| 9 | partitioning defective 3 homolog (par-3) | 174 | Hs.131489 | NM_019619 | Forward GTCCTTCGAGCAAATATGCCTCT  Reverse TAGGACTCCCAGCAGTGTTCTG  Probe TGTTCGACGCAGTAGTGACCCAGCTCTAA |
| 10 | partitioning defective 6 homolog alpha (par-6) | 153 | Hs.112933 | NM_016948 | Forward AAGCGGGCAGAAGCTGACT  Reverse CTGAGGAAACCTGGCGGAA  Probe TGCGGCCAGTGGCACCCC |
